# Supplementary material for: Xputer: bridging data gaps with NMF, XGBoost, and a streamlined GUI experience
Source: Front Artif Intell. 2024 Apr 24;7:1345179. doi: 10.3389/frai.2024.1345179 (PMC11076752; doi:10.3389/frai.2024.1345179)
Supplement: Supplementary file 1 [file Data_Sheet_1.PDF]

Xputer: Bridging Data Gaps with NMF, XGBoost, and a Streamlined GUI Experience

Supplementary methods

Procedure for configuring the Xputer within a Python-based environment:

Installation

1. *Install Anaconda:* Download the latest version of Anaconda from www.anaconda.com. Install by double-clicking the downloaded installer. When the installer asks for the installation type, choose "Just Me".
2. *a) To Install Xputer on Windows*:
   - Locate the "Anaconda PowerShell Prompt" using the Windows search function.
   - Open it and input the following command.
   - `pip install xputer`
   - Hit "Enter" to begin the installation of Xputer and its associated packages.

   b) To Install Xputer on MacOS

   - Locate the "Terminal" application and open it.
   - Prior to installing the package, initiate the base conda environment.
   - Do this by entering the command below and then pressing "Enter":
   - `conda activate`
   - "base" should be prepended.
   - Type the following command and press "Enter" to install Xputer:
   - `pip install xputer`
   - To deactivate the conda environment use the following command:
   - `conda deactivate`

   C) Platform-independent alternative

   - Launch Anaconda Navigator
   - From the navigator launch JupyterLab. It will open a browser window.
   - Click on Terminal from the browser window.
   - Type the following command in Terminal/PowerShell window:
   - `pip install xputer`
   - Press "Enter" to install Xputer.

Run Xputer

1. *Run on Windows:*
   - Locate the "Anaconda PowerShell Prompt" using the Windows search function.
   - Open it and input the following command.
   - `python -m xputer`
   - Press "Enter" to open the Xputer GUI

2. *Run on MacOS:*
   - Locate the "Terminal" application and open it.
   - Prior to installing the package, initiate the base conda environment.
   - Do this by entering the command below and then pressing "Enter":
   - `conda activate`
   - "base" should be prepended.
   - To run Xputer, type the following command and then press "Enter":
   - `python -m xputer`
   - To deactivate the conda environment use the following command:
   - `conda deactivate`

3. To run on platform-independent alternative

- Launch Anaconda Navigator
- From the navigator launch JupyterLab. It will open a browser window.
- Click on Terminal from the browser window.
- Type the following command in Terminal/PowerShell window:
- `python -m xputer`
- Press "Enter" to run Xputer GUI.

Use Xputer from the GUI

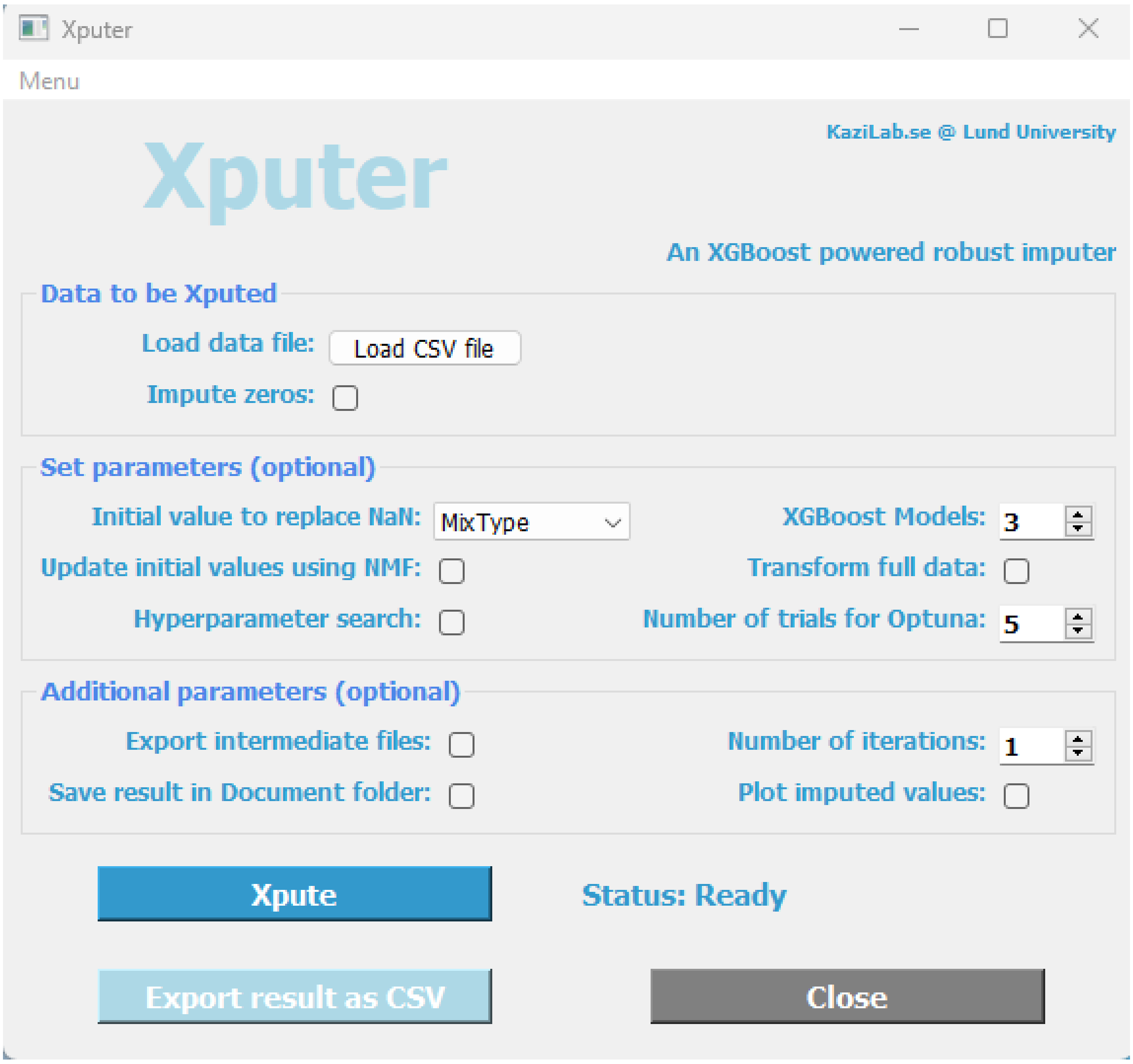

1. For imputation to proceed, the data should be supplied in a CSV format. In this format, the first column should list the sample names, while the top row should enumerate the feature names. See the example below:

Sample	Feature 1	Feature 2	Feature 3	Feature 4	Feature 5	Feature 6	Feature 7	Feature 8
Sample 1	Data	Data	Data	Data	Data	Data	Data	Data
Sample 2	Data	Data	Data	Data	Data	Data	Data	Data
Sample 3	Data	Data	Data	Data	Data	Data	Data	Data
Sample 4	Data	Data	Data	Data	Data	Data	Data	Data

2. To load the data file in CSV format, click on "Load CSV file", locate the file, and press open.
3. Mouse over parameters to get inline help.
4. Click on "Xpute" to run imputation.

Optional options:

1. To impute zeros, select the "Impute zeros" checkbox.
2. You can replace initial NaN values using one of three methods from the dropdown list.
3. The "XGBoost Models" option, used for averaging the final imputation, allows a range from 3 to 9 models.
4. If "Update initial values using NMF" is selected, the data matrix will be factorized and reconstructed using either NMF or SVD (chosen if a negative value is present). Only the NaN values will then be replaced by the transformed values.
5. If "Transform full data" is selected, the data matrix will be factorized and reconstructed using either NMF or SVD (if a negative value is present). The fully transformed data will then be utilized for XGBoost prediction.
6. Checking "Hyperparameter search" will engage Optuna for XGBoost hyperparameter optimization. The "Number of trials for Optuna" setting determines the number of trials for this search.
7. If "Export intermediate files" is checked, Xputer will save all intermediate files to the "Document" folder.
8. "Number of iterations" lets you adjust the count of XGBoost iterations.
9. If "Save result in Document folder" is checked, results will be saved automatically in that folder.
10. Selecting "Plot imputed values" will generate and save both density plots and dot plots.

Advanced options:

To use in the pipeline:

```
from xputer import xpute

xpute function returns the imputed dataframe

imputed_df = xpute(df,
                   impute_zeros=False,
                   pre_imputation='MixType',
                   xgb_models=3,
                   mf_for_xgb=False,
                   use_transformed_df=False,
                   optuna_for_xgb=False,
                   optuna_n_trials=5,
                   n_iterations=1,
                   save_imputed_df=False,
                   save_plots=False,
                   test_mode=False)
```

or

```
from xputer import Xpute
xpute = Xpute(impute_zeros=False,
              pre_imputation='MixType',
              xgb_models=3,
              mf_for_xgb=False,
              use_transformed_df=False,
              optuna_for_xgb=False,
              optuna_n_trials=5,
              n_iterations=1,
              save_imputed_df=False,
              save_plots=False,
              test_mode=False)
imputed_df = xpute.fit(df)
```

df: pandas DataFrame with index (sample names) and header (features).

impute_zeros: Boolean.

pre_imputation: 'MixType', 'ColumnMean', 'KNNImputer'.

xgb_models: Integer, between 3 and 9.

mf_for_xgb: Boolean.

use_transformed_df: Boolean.

optuna_for_xgb: Boolean.

optuna_n_trials: Integer, between 5 and 50.

n_iterations: Integer, between 1 and 9.

save_imputed_df: Boolean.

save_plots: Boolean.

test_mode: Boolean.

Use independent modules:

```
from xputer import preprocessing_df
```

preprocessing_df returns a clean_df, an encoded_df, and a preimputed_df

```
clean_df, encoded_df, preimputed_df = preprocessing_df(df,
                                            impute_zeros=False,
                                            pre_imputation='MixType',
                                            test_mode=False)
```

```
from xputer import cnmf
```

cnmf function returns two dataframes, NaN_imputed_by_NMF and Fully_transformed_df, when provided encoded_df and preimputed_df from preprocessing_df.

```
NaN_imputed_by_NMF, Fully_transformed_df = cnmf(encoded_df, preimputed_df)
```

```
from xputer import run_svd
```

run_svd function is similar to cnmf except here it initiates SVD instead of NMF.

```
NaN_imputed_by_svd, Fully_transformed_df = run_svd(encoded_df,
                                                   preimputed_df)
```
